# Exposed Pulps

**Published:** 1875-11

**Authors:** Wm. C. Wardlaw

**Affiliations:** Augusta, Ga.


### ARTICLE IV.

### *Exposed Pulps.*

WM. C. WARDLAW, D. D. S., AUGUSTA, GA.

I had prepared a paper upon the treatment of "exposed pulps" with my method of "capping" them, when your admirable editorial upon the subject, in the May No. of the *Register*, appeared. The principles of treatment are there so clearly and satisfactorily set forth, (the reader is particularly referred to it,) that it will not be necessary, for my present purpose, that I should do more than briefly recapitulate them in this article, in which I wish to direct attention to my mode of "capping nerves."

The first indication of treatment, of course, is to remove and permanently exclude all irritants.

This can only be done thoroughly, by a free and full opening-up of the cavity, so that direct access may be had to every part of it. The condition of the pulp itself will then suggest the therapeutic treatment demanded. If inflamed or congested, sedatives and astringents will be required; if suppurating, Oakley Coles treatment of pepsin is good enough. A systemic regimen may have to be instituted to meet a malarial mercurial, or syphilitic cachexia. But, in a healthy system, the main thing is to protect the pulp from irritation, and nature will come to the rescue, and very soon restore it to a normal condition.

Having, then, freed the pulp from all external irritants, and made such topical application as may be necessary, the next step is to secure this condition, to await the kind offices of nature, or, as the basis for a permanent operation.

With the pulp in readiness, what are the requirements of a nerve-capping? The material must be un-irritating, a non-conductor, accurately adapted to the point of exposure, and hermetically sealing the orifice to the pulp.

You say, "these requirements are easily understood; but the substance to supply them completely is not yet obtained."

I claim that I have such a substance, and that my mode of using it is superior to any of the many methods of "capping" pulps, that I've seen recommended. I do not say that it is not used by others, but I claim that, with myself, it is original and that it has been very successful in my hands. Dr. King's method of the batter of oxide of zinc and creosote with the os-artificial is good. Dr. Francis' method of note paper and balsam of fir, with os-artificial, is good; the method of the egg membrane, I have used, with happy results, isinglass, court-plaster and os-artificial; but none of these modes completely fill the bill.

My material is the common pink base-plate gutta-percha dissolved in chloroform, in conjunction with os-artificial. Notice, the gutta-percha is the base-plate and has its own peculiar advantages.

With the cavity carefully prepared, the rubber dam in position, and all things in readiness, a drop of the solution taken up with an instrument or on a pellet of cotton, is to be applied to the point of exposure.

The chloroform very rapidly evaporates, leaving a thin pellicle covering on the exposed pulp. The evaporation which is very prompt, may be hastened with the air syringe, and the coating may be made thicker, if desirable, by a second application.

Upon this flooring an os-artificial filling is to be placed, which will afford a firm foundation for a gold filling.

We thus have our pulp protected by a durable material which, applied in a plastic condition, has smoothly and accurately conformed itself to the surface of the pulp, is unirriatting and a non-conductor, and is firmly adherent to the floor of the cavity.

With this condition of things preserved, will not nature attend to the balance? We may safely trust her. Very slight co-operation is all she needs. I have great faith in the "viz medicatrix naturæ." Nearly any pulp, however inflamed, short of suppuration, will return to a normal condition if secured from irritation. I fear many pulps are dosed to death with escharotic applications.

The following peculiar characteristic properties particularly recommend this solution. The gutta-percha is so promptly soluble that it can be prepared upon the moment and at nominal cost. It evaporates very rapidly, acquiring the desired consistency directly. It contains so much earthy matter as to form a stratum of appreciable thickness. It is very adhesive, sticking closely to the walls of the cavity. Its pink color renders it readily visible wherever placed,—an important consideration. It is a perfect non-conductor. The chloroform is soothing to the pulp and sensitive dentine. It can be made so thick as to be easily applied to an upper tooth even. And, finally, being insoluble in the chloride of zinc, it cuts off the usually severe pain following the application of the os-artificial. This last, I regard as of great moment, for I believe many pulps succumb to the escharotic effect of the chloride of zinc, which otherwise would have survived.

I had tried, several years ago, to use, in a similar manner, the white gutta-percha solution, obtained at the dental depots, but abandoned it because it evaporated so slowly, it left too thin a film, its color made it impossible to know what was being done with it, and, it was so fluid that it could not be used in upper cavities, except with great difficulty.

I have so much satisfaction in the daily use of this solution for this, and a variety of purposes, that I beg my professional brethren who may have a prejudice against it, or in favor of something else, to just try it for a few times, and I think they will involuntarily exclaim "Eureka." I wanted to give in detail some of these other uses, but the length of this article admonishes me against it. I will merely mention some of them.

I use it, as a temporary filling in sensitive cavities, as a covering over arsenic in shallow cavities, as a temporary expedient in cavities where, for the want of light, time, or other reason, I can't make a thorough examination; in the aching teeth of timid children ("*ne plus ultra*,") in con junction with os-artificial, over nearly exposed nerves, to intercept the chloride of zinc; to protect os-artificial fillings against moisture; to stop a leak through an accidental puncture in the rubber dam; upon a conical tooth to prevent the dam from slipping off, etc., etc. It is in fact, my "Man Friday," without which I cannot get along.

The first I ever saw of the solution, Dr. E. W. Harker was using it for the protection of his os-artificial fillings against moisture, and to him belongs the credit, so far as I know of its introduction in dentistry.—*Dental Register.*

